# Outbreak of *Salmonella enterica* subsp. *enterica* Serovar Napoli on a Dairy Cow Farm

**DOI:** 10.3390/ani15010079

**Published:** 2025-01-02

**Authors:** Matteo Ricchi, Anita Filippi, Erika Scaltriti, Martina Tambassi, Stefano Pongolini, Luca Bolzoni, Alice Prosperi, Camilla Torreggiani, Medardo Cammi, Alessandro Chiatante, Norma Arrigoni, Elisa Massella, Andrea Luppi, Chiara Garbarino

**Affiliations:** 1 Experimental Zooprophylactic Institute of Lombardy and Emilia Romagna (IZSLER), “Bruno Ubertini”, Diagnostic Section of Piacenza, Italy Via Strada Della Faggiola 1, 29027 Podenzano, PC, Italy; anita.filippi@izsler.it (A.F.); alice.prosperi@izsler.it (A.P.); camilla.torreggiani@izsler.it (C.T.); norma.arrigoni@izsler.it (N.A.); elisa.massella@izsler.it (E.M.); andrea.luppi@izsler.it (A.L.); chiaraanna.garbarino@izsler.it (C.G.); 2 Risk Analysis and Genomic Epidemiology Unit, Experimental Zooprophylactic Institute of Lombardy and Emilia Romagna (IZSLER), “Bruno Ubertini”, Strada dei Mercati, 13/A, 43126 Parma, Italy; erika.scaltriti@izsler.it (E.S.); martina.tambassi@izsler.it (M.T.); stefano.pongolini@izsler.it (S.P.); luca.bolzoni@izsler.it (L.B.); 3 Independent Researcher, 29100 Piacenza, Italy; docmedo@libero.it; 4 Local Health Authority of Piacenza, Via Antonio Anguissola 15, 29121 Piacenza, Italy; alessandro@chiatante.it

**Keywords:** *Salmonella* Napoli, outbreak, dairy cows

## Abstract

*Salmonella* is diffused worldwide, and *Salmonella enterica* subsp. *enterica* is spread worldwide with many serovars associated with the infection of domestic bovines. The most spread are *S*. Dublin, *S*. Typhimurium and *S*. Infantis. *S.* Napoli is, however very rarely reported in domestic ruminants. Here, we report an outbreak of *S*. Napoli on a dairy cow farm in Northern Italy (Piacenza). A total of 18 *S*. Napoli isolates were recovered from aborted fetuses, feces, tissues and environmental samples. Whole genome sequencing suggested that all isolates belonged to the same cluster. After the application of stringent biocontainment and biosecurity measures, no further cases were reported. However, four months after the first case, the serovar was still isolated in environmental samples, underlying the importance of adopting the correct biosecurity and biocontainment measures in order to prevent the circulation and transmission of *Salmonella* within the farm.

## Simple Summary

*Salmonella* is considered one of the most widespread pathogens in both humans and animals worldwide. Among different *Salmonella*, some serovars are generally associated with salmonellosis in calves and adult cows, causing mild to severe illness. However, other serovars can be considered as infective and are able to cause diseases in both humans and animals. This case report showed that *S*. Napoli, a serovar rarely isolated from bovines, caused an outbreak in a dairy cow herd. The genomic analyses of all recovered *S*. Napoli isolates suggested the spread of a single infective event by a clone within the herd. Increased biosecurity and biocontainment measures appeared to have helped to contain the spreading of the disease even if environmental contamination persisted.

## 1. Introduction

*Salmonella* are rod-shaped, gram-negative bacteria belonging to the *Enterobacteriaceae* family [1]. Two species are currently recognized within the *Salmonella* genus: *Salmonella enterica* and *Salmonella bongori* [2]. *Salmonella enterica* is further subdivided into six subspecies which include multiple antigenic variants classically defined as serovars [3,4].

*Salmonella* infections in bovines are commonly associated with serovars belonging to the *Salmonella enterica* subsp. *enterica* species (hereafter defined as “*S.*” or “*Salmonella*”) [5,6]. The effects of *Salmonella* infection in cattle can include: (a) gastroenteritis associated with diarrhea, dehydration, lethargy, dysorexia and fever; and (b) systemic infections sometimes associated with septicemia and high mortality rates. Moreover, in pregnant cows, infection with certain serovars, such as *S.* Dublin and *S.* Typhimurium, may lead to abortion or stillbirth [7]. Less common clinical signs are reduction in milk production and arthritis [7,8].

One of the main *Salmonella* serovars associated with bovine infections is *S*. Typhimurium, known to infect a wide range of hosts, including cattle [5,6]. This serovar is often associated with gastroenteritis and systemic infections in bovines [6]. Another serovar typically associated with bovine diseases is *S*. Dublin [5], which is known to cause severe systemic infections with reproductive diseases and pneumonia as common manifestations of infection in calves [5,6].

The prevalence of *Salmonella* serovars in bovine populations can vary significantly among different countries and regions. For example, a recent study in the USA [9] reported that, of the nearly 5000 field isolates analyzed, *S*. Dublin accounted for almost 23% of the total, followed by *S*. Cerro (around 16%), *S*. Newport (14%), *S*. Montevideo and *S*. Kentucky (both 8%) and *S*. Typhimurium (4%). On the other hand, the latest available report by EFSA underlined that the serovars most frequently recovered from European bovines were *S*. Typhimurium (35.8% of total sampling units in bovines), *S*. Dublin (34.3%) and *S*. Infantis (5.8%) [10]. In Italy, the latest report by the Italian Reference Centre for Salmonellosis [11] reported that, among bovine field isolates (340), the most spread serovars were *S*. Typhimurium and its monophasic variant, together accounting for almost 70% of the total, followed by *S*. Dublin (19%).

*S*. Napoli was very rarely isolated from cattle [10]. It is usually isolated from environmental specimens [11]. Contaminated environments and wildlife animals are considered the primary sources of infection for this serovar, as recently suggested in wild boars in Italy [12]. However, a study including only isolates from Southern Italy reported the isolation of *S.* Napoli also from buffaloes [11]. Furthermore, over the last ten years, this serovar has emerged as an invasive pathogen responsible for human diseases in Italy [13,14] and neighboring countries, such as Switzerland and France [10,15].

In this paper we report an outbreak caused by *S.* Napoli that occurred on a dairy cattle farm in the province of Piacenza, Northern Italy.

## 2. Materials and Methods

### 2.1. Farm

The farm is located in the province of Piacenza, in the Emilia Romagna region in Northern Italy. At the time of sampling, the herd consisted of 310 Holstein Friesian cows. The herd was almost exclusively dedicated to milk production but four animals intended for beef production were also housed. Moreover, there were 80 heifers (ages ranging from six to 24 months) and 62 calves (under six months of age). No other animals were housed in the farm except for one dog. However, the presence of synanthropic animals, including mice, cannot be excluded. About 40–50 milking cows present in the herd were cows in the dry period, while the remainder were in lactation. The farm practiced artificial insemination and no introduction of externally-born animals had occurred since 2018. The farm was a free-stall farm area, where the animals were housed and divided based on their lactation conditions and age. The farm is located in the Pianura Padana, an area characterized by flat terrain with no hills or mountains, and it is not encircled by physical barriers, such as fences. However, a ditch partially delineates the boundaries of the property. The farm is surrounded by fields frequented by wildlife. Coypus (*Mycocastor coypus*), wild boars (*Sus scrofa*), foxes (*Vulpes vulpes*), roe deer (*Capreolus capreolus*) and wolves (*Canis lupus*) are frequently observed and have been known to occasionally enter the perimeter of the property.

### 2.2. Outbreak Description and Subsequent Sampling

At the beginning of December 2022, our laboratory received two bovine fetuses aborted on the farm. The samples were carried to our laboratory because a control plan related to the survey on abortion agents was in force in the Emilia Romagna region (Italy). The fetuses underwent a complete necropsy. There was direct isolation of *Brucella* spp., as well as PCR analyses for the detection of *Leptospira* spp., Q fever, *Chlamydia* spp., *Neospora caninum*, *Schmallenberg virus,* and PCRs to detect all Pestiviruses (PCR Pan Pestivirus), *Mycoplasma* spp., *Bovine herpesvirus*-1,2 and 4 (BHV-1,2 and 4) from a pool of *viscera* (liver, spleen, kidney, thymus gland and lungs), brain and cerebellum and muscle (both skeletal and cardiac muscles) had negative results. Notably, bacteria belonging to the genus *Salmonella* spp. were directly isolated.

*Salmonella* spp. was subsequently isolated from the organs of a male calf seven days old (Table 1), and even in this case no other infectious agents were detected.

On the 19 December 2022, a fecal specimen from a sick cow with diarrhea was analyzed according to ISO 6579 part 1 [16] and was found positive for *Salmonella* spp.

On the 2 January 2023, a second dead female calf (11 days old) was delivered to our laboratory, and *Salmonella* spp. was isolated from different organs (spleen, liver and lungs). In this case as well, the PCR results were negative for all previously mentioned infectious agents.

On the 17 January 2023, a veterinary inspection was conducted by the *local health authority* in order to assess biosecurity and biocontainment measures in place in the farm, according to the list included in the reference [17] and in the current manual for managing *Salmonella* infection in milking cows, a product of a specific research project aimed at managing salmonellosis cases in dairy cows (https://www.izsler.it/wp-content/uploads/sites/2/2023/09/Manuale-Operativo-Salmonellosi-nellallevamento-Bovino.pdf, accessed on 10 December 2024). During the inspection, non-randomized environmental sampling was carried out from different sources (Table 2) for a total of 10 samples, and one fecal specimen, from a single sick milking cow with fever was also taken (Table 1).

A second round of non-randomized environmental samplings (eight samples) was carried out on March 2023 in some areas of the farm during a second visit (Table 2). The frequency of positive samples between the first and second visits were compared using the Fisher exact test (MedCalc Software Ltd. Fisher exact probability calculator. https://www.medcalc.org/calc/fisher.php, Version 23.0.6; accessed on 7 November 2024).

### 2.3. Cultural Methods and Serotyping

All *Salmonella* spp. environmental isolates, as well as those from fecal specimens, were isolated according to the ISO 6579 part 1 [16]. The only exceptions were the isolates recovered from the calves and the fetuses, where a direct streaking of organs was carried out on both non-selective (blood agar, IZSLER internal production, recipes available upon request) and selective (Gassner, Biolife, Milan, Italy) media. *Salmonella* isolates were identified through the EnteroPluri–Test Gallery (Liofilchem, Teramo, Italy) after isolation on selective media. Serotyping was then carried out according to the ISO 6579 part 3.

### 2.4. Whole Genomic Sequencing (WGS)

Genomic DNA was extracted using the Maxwell(R) HT 96 gDNA Blood purification kit (Promega, Madison, WI, USA) and was subjected to genomic libraries preparation using the Illumina^®^ DNA Prep (M) Tagmentation kit (Illumina, San Diego, CA, USA). Paired-end reads (150 × 2 bp) were obtained using an Illumina Nextseq platform (Illumina, San Diego, CA, USA).

Raw reads were trimmed for quality using Trimmomatic [18] and then assembled with Unicycler version 0.5.1 [19]. Data are available at the following link: https://www.ebi.ac.uk/ena/browser/view/PRJEB74287, accessed on 10 December 2024. In-silico antimicrobial resistance (AMR) and virulence gene analyses were performed, respectively, using ResFinder, PointFinder [20] and the Virulence Factor Database (VFDB) Analyser [21].

A core genome Multi-locus Sequence Typing (cgMLST) analysis based on the En-terobase scheme (https://enterobase.warwick.ac.uk/, accessed on 10 December 2024) was performed on the *S*. Napoli isolates of this study and *S*. Napoli genomes belonging to our genomic surveillance da-tabase, which includes *Salmonella* genomes of human, animal and environmental origin isolated in the Emilia-Romagna region from 2021 to 2024 as part of the Regional *Salmonella* Surveillance Activities. A cgMLST analysis was visualized in a Minimum Spanning Tree (MST) produced with Bionumerics 7.6 (Biomerieux, Bagno a Ripoli (FI), France).

A core Single Nucleotide Polymorphism (SNP) analysis was performed with CFSAN SNP Pipeline ver. 2.1.1 [22] with default settings available at https://snp-pipeline.readthedocs.io/en/latest/ (accessed on 10 December 2024) using the 2022-421902-001 isolate as reference, while SNP phylogeny was inferred using a Maximum Likelihood (ML) algorithm with 100 bootstraps with PhyML [23]. The obtained tree was visualized using Figtree v 1.4.4 (http://tree.bio.ed.ac.uk/software/figtree/, accessed on 10 December 2024). Outgroups (2022-341907-001-01, 2022-281858-004-01, 2022-266440-006-01 and 2022-289262-003-01) were isolates from human cases of salmonellosis, taken from the Regional Salmonella Surveillance of the Emilia-Romagna region (Northern Italy).

## 3. Results

### 3.1. Sampling, Environmental Control and Farm Visit Results

*Salmonella* spp. isolated from two fetuses, a seven-day-old male calf and an 11-day-old female calf, both with septicemia and fecal samples collected from the diseased cows, were identified as *S.* Napoli (Table 1). No further infectious agents were detected.

Following the first visit by the local health authority, non-randomized environmental sampling was carried out and *S*. Napoli. isolates were recovered from different sources (Table 2) in nine out of ten environmental samples.

The most significant issues identified during this visit were the presence of a ditch surrounding the farm, the dirtiness of the calving pen and the presence of synanthropic wild animals. During this visit, the farmer received feedback, which included the following recommendations: to improve environmental and equipment hygiene (including devices for food preparation and distribution), to increase the frequency of the cleaning and disinfection of the scrapers used to remove manure from paddocks and the number of passages of these scrapers and to pay particular attention to the hygiene of the delivery box and the area where sick animals were housed. Furthermore, actions for increasing biosecurity were recommended: to employ dedicated tools and materials for the management of adult animals and calves, to prevent access to housing areas by other domestic (dog) and synanthropic (mice, pigeons, and others) animals by undertaking pest control procedures and, finally, to adopt strict hygiene measures during milking.

During a second inspection, *S*. Napoli was still isolated, albeit sporadically (three positives out of eight total samples, see Table 2). Despite the detection of *Salmonella* following the second environmental sampling, no further clinical or subclinical cases were identified in animals.

The frequency of positive samples between the first and second visits showed a significant difference (Fisher exact test *p* = 0.04).

### 3.2. Genetic Characterization of S. Napoli Isolates

WGS analysis revealed that all 18 *S*. Napoli isolates listed in Table 1 and Table 2 belong to Sequence Type (ST) 474. Moreover, all *S*. Napoli isolates were genetically closely related, with SNPs’ pairwise distances ranging from 0 to 6 SNPs and a maximum of 3 SNPs in single linkage clustering.

Based on cgMLST analysis, the genomes of the *S*. Napoli from the farm were not genetically related (see separate blue cluster in Supplementary Figure S1) to any *S*. Napoli genome belonging to our database, which includes 152 genomes of *S*. Napoli of human, animals, such as wild boar, chicken, porcupine, hedgehog, badger, grey partridge, wolf and enviromental samples not origin isolated in the Emilia-Romagna region from 2021 to 2024. The closest genomes from isolates of human origin were distant, from 41 to 57 alleles, suggesting no genetic relationship between them and the *S*. Napoli isolates from the farm. The aforementioned human isolates were also used as an outgroup in phylogenetic analysis.

**Figure 1:**
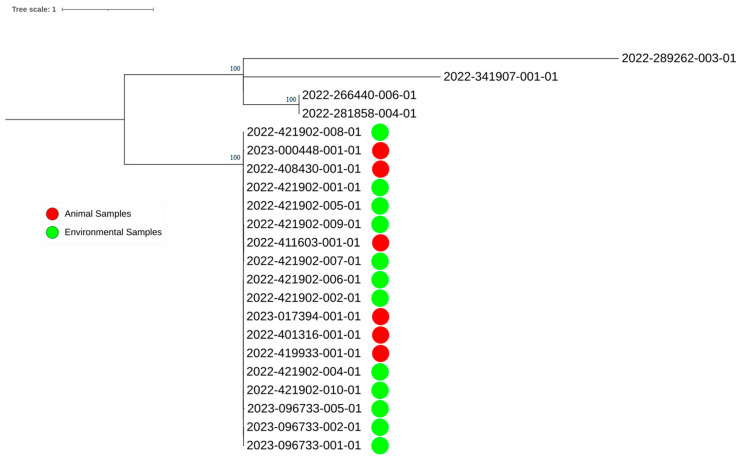
Phylogenetic tree showing all *S*. Napoli isolates recovered in the farm and four other human strains from the same region and year of isolation used as outgroup. In red: animal samples and in green: environmental samples. Bootstrap values <59 are reported at the nodes.

The maximum-likelihood tree showed that all genomes of the *S*. Napoli isolated belong to the same clade (Figure 1). These results demonstrated the clonality of the contamination [24], suggesting the spread of this was due to a single clone within the herd. The outgroup genomes displayed genetic distances ranging from 77 to 353 SNPs compared to the *S*. Napoli isolates studied, confirming that they were not genetically related.

We next performed an in-silico AMR and virulence gene analysis on the *S*. Napoli genomes from the farm and the genomes used as the outgroup in the phylogenetic analysis. The aac(6′)-Iaa gene conferring resistance to amikacin and tobramycin was found in all the *S*. Napoli analyzed (Supplementary Table S1). No other AMR genes or point mutations conferring AMR were detected except for the blaTEM-1B in an outgroup genome. Furthermore, the outbreak isolates did not carry any additional virulence genes compared to the outgroup (Supplementary Tables S1 and S2).

## 4. Discussion

*Salmonella enterica* can impact the health status of bovines, causing diseases ranging from mild to severe [1,5]. However, it should be remembered that the severity of *Salmonella* spp. infections can be influenced by factors such as the specific serovar involved, the age and health status of the animal, as well as any stress factors, such as transportation or changes in diet [5].

Contaminated food, water and contact with other infected animals are common sources of *Salmonella* transmission in livestock [1,17]. Infected cows that recover from clinical illness may become carriers, without showing evident signs of disease.

In the outbreak described here, the serovar recovered was *S*. Napoli. This serovar was very rarely found in bovine herds. Few anecdotal cases without the description of the circulation of the pathogen within the farm have been reported for this specific serovar in bovines [10] and similar-housed animals, such as buffaloes [25].

The genomic evidence collected seems to support the hypothesis that a single clone of *S*. Napoli spread throughout the farm. All the isolates recovered, both from animals and environmental sources, were closely genetically related, with a number of genetic differences observed (0–6 SNPs) consistent with the hypothesis of the spread of a single clone. Notably, the isolates recovered from this outbreak were not genetically related to other *S.* Napoli isolates of the same ST from humans, animals and environment within the genomic database of the Regional *Salmonella* Surveillance of the Emilia-Romagna region. However, more samples collected over time would be required to confirm this. In fact, we recognize our analysis does not preclude the possibility that multiple infection events may have occurred, with only one clone disseminating the infection within the farm.

Because of the above-mentioned limits and according to the available information on the epidemiology of *S*. Napoli [12,26,27,28], we can only speculate that the most plausible route of infection was through close proximity to wild animals, such as wild boars, roe deer, coypus, carnivores (foxes or wolves) and also wildlife birds [27]. In fact, the farm was not separated by fences or other physical barriers. It was merely surrounded by a ditch, which could have allowed direct and indirect contact between cows and wild animals, such as through feces from wild animals. It is also possible that the presence of an automatic feed dispenser, which can be remotely controlled, may have attracted wild animals, thus representing an additional risk factor for the entry of the pathogen onto the farm. Furthermore, this dispenser may have contributed to the spread of the pathogen within the farm. Indeed, the detection of *S*. Napoli in wild animals [26] and the co-circulation of the same *Salmonella* clones among wild animals and farmed bovines [11] have been already documented in the Emilia-Romagna region (where the contaminated farm is located).

Notably, “in silico” analyses of the whole genomes of the sequenced isolates did not reveal any particular virulence factor that would differentiate this clone from others of the same serotype and justify the ability of this clone to sustain infection or persist in the environment. However, it is well known that *S*. Napoli is virulent in humans [25]. On the other hand, any outbreak is the result of complex interactions between the pathogen, the environment and the host. In our case, the conditions resulting from this interaction could have been favorable for the pathogen and, even though no specific virulence factor was found in the genomes of our isolates and *S*. Napoli is not a serovar normally associated with infection of domestic cattle, this pathogen was able to maintain an outbreak in cows. In particular, the recovery of the same pathogen in several places on the farm and in animals of different ages indicated that the absence of robust biocontainment conditions on the farm could have played a role in the pathogen spread. In this respect, although a significant difference in the number of positive environmental samples was obtained between the two sampling periods, before and after the recommendations, the limited number of environmental samples collected, the non-randomized protocol used and the observation that positive samples were found in areas housing animals of different ages, do not allow any definitive conclusions to be drawn regarding the effectiveness of the biocontainment measures applied by the farmer and based on the health management plan of the manual for the management of *Salmonella* infection in dairy cows (available at https://www.izsler.it/wp-content/uploads/sites/2/2023/09/Manuale-Operativo-Salmonellosi-nellallevamento-Bovino.pdf, accessed on 10 December 2024). Conversely, the control plan related to the abortion survey is still in place in the Emilia-Romagna region and the farm has been continuously monitored for infectious diseases, but no further *Salmonella* positive infection linked to this farm, both in cows and humans, has been reported. These last observations suggest that the biosecurity measures and the suggestions provided to the farmer, such as the boiling of raw milk prior to consumption and the wearing of appropriate footwear and attire upon entering the residence [17], could have limited the spreading of the pathogens within animals. Some of these suggestions have also been included in the very recent “Protocol for the management of a salmonellosis outbreak in dairy cow farms” licensed by the Italian Reference Centre for Salmonellosis on May 2024 and available at https://www.izsvenezie.it/protocollo-gestione-focolaio-salmonellosi-allevamenti-bovine-latte/, accessed on 10 December 2024.

*S*. Napoli has been isolated in Italy in recent years and in other neighboring countries [10,13,14]. Notably, this serovar appears to be more prevalent in environments where wild animals, such as wild boars, are present [12]. However, in this instance, the serovar was identified in a domestic environment. Interestingly, in the same macro-area where the farm is located, a few years ago, an unexpected increment in *S*. Napoli in human cases was observed [28]. However, the reasons behind this increment still remain unexplained [15], but the isolation of the *S*. Napoli serovar was reported from different foodstuffs [15]. The isolation of *S*. Napoli, a serovar with a low degree of host specificity, in a dairy herd, represents a potential risk to human health, particularly given the lack of clarity surrounding several epidemiological aspects.

## 5. Conclusions

The present study highlights the potential, even for a *Salmonella* serovar not normally recovered in dairy herds, to cause serious and difficult-to-manage outbreaks. The epidemiological aspects of the infection sustained by *S*. Napoli remain not clearly understood and further studies should be carried out in order to investigate the potential for cross-species transmission between domestic and wild animals, with the final aim of improving the effectiveness of the biocontainment and biosecurity measures that should be taken at farm level in order to control these outbreaks.

## Figures and Tables

**Table 1:** Characteristics and time of collection of the *S*. Napoli positive animals analyzed. For each sample, ID, source of isolation, data of sampling and results of clinical/anatomical examination are reported. All samples resulted positive for *Salmonella* spp., which was further identified as *S.* Napoli.

ID	Source of Isolation	Date of Sampling	Anatomopathological Lesions
401316	Foetus	5 December 2022	None.
408430	Foetus	9 December 2022	None.
411603	Calf, 7 days old	13 December 2022	Intestinal wall thickening and yellowish fluid contents; mesenteric lymph nodes hyperplasia; mild splenomegaly.
419933	Faeces from milking cow	19 December 2022	/
448	Calf, 11 day old	2 January 2023	Pulmonary congestion and suppurative pneumonia; myocardial petechiae; widespread catarrhal/catarrhal-hemorrhagic enteritis in the small and large intestine.
17394	Faeces from milking cow	17 January 2023	/

**Table 2:** List of environmental samples collected in the farm. For each sample, number of visit by local health authority, ID, source of isolation, result of *Salmonella* spp. isolation and data of sampling are reported. All isolates recovered were further identified as *S.* Napoli.

Visit	ID	Source of Isolation	Result	Data of Sampling
1st	421902/1	Waiting room	Positive	20 December 2022
1st	421902/2	Calves’ paddock	Positive	20 December 2022
1st	421902/3	Manure scraper of the 1st lactating group (Sample 1)	Negative	20 December 2022
1st	421902/4	Manure scraper of the 2nd lactating group (Sample 1)	Positive	20 December 2022
1st	421902/5	Manure scraper of the 1st lactating group (Sample 2)	Positive	20 December 2022
1st	421902/6	Manure scraper of the 2nd lactating group (Sample 2)	Positive	20 December 2022
1st	421902/7	Manure scraper of the dry cow area, delivery room and post-partum room (pooled sample)	Positive	20 December 2022
1st	421902/8	Manure scraper of the 1st group (Sample 3)	Positive	20 December 2022
1st	421902/9	Manure scraper of the 2nd group (Sample 3)	Positive	20 December 2022
1st	421902/10	Manure scraper of waiting room for milking parlor	Positive	20 December 2022
2nd	96733/1	Manure scraper of facilities for sick animals, cows in last period before and post calving	Positive	27 March 2023
2nd	96733/2	Manure scraper of the 1st lactating group (Sample 1)	Positive	27 March 2023
2nd	96733/3	Manure scraper of the 2nd lactating group (Sample 1)	Negative	27 March 2023
2nd	96733/4	Manure scraper of the 1st lactating group (Sample 2)	Negative	27 March 2023
2nd	96733/5	Manure scraper of the 2nd lactating group (Sample 2)	Positive	27 March 2023
2nd	96733/6	Calving Pen	Negative	27 March 2023
2nd	96733/7	Calves’ paddock 1 (preweaning animals)	Negative	27 March 2023
2nd	96733/8	Calves’ paddock 2 (postweaning animals)	Negative	27 March 2023

## Data Availability

Genomic data are available at the European Bioinformatics Institute (EMBL-EBI) under study accession n. PRJEB74287D. further data are available upon request to the auhtors.

## Supplementary Materials

The following supporting information can be downloaded at: https://www.mdpi.com/article/10.3390/ani15010079/s1.
