# Supplementary figures and images for: Outbreak of Salmonella enterica subsp. enterica Serovar Napoli on a Dairy Cow Farm
Source: Animals (Basel). 2025 Jan 2;15(1):79. doi: 10.3390/ani15010079 (PMC11718894; doi:10.3390/ani15010079)

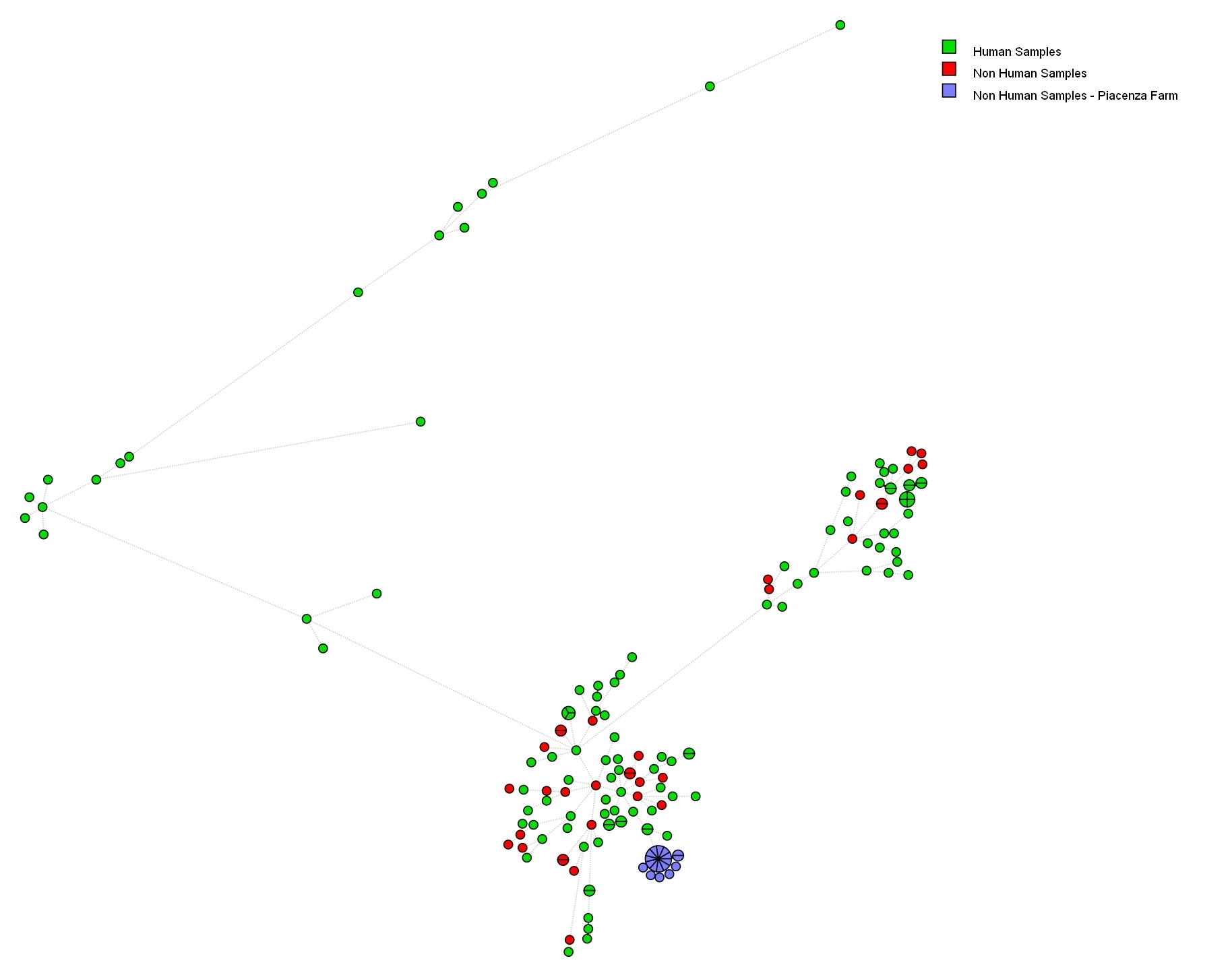

Supplement: Supplementary file 1 [file animals-15-00079-s001.zip › Supplemental Figure S1.jpg]
